# Electrospun poly(l-lactide-*co*-dl-lactide) nanofibrous scaffold as substrate for *ex vivo* limbal epithelial cell cultivation

**DOI:** 10.1016/j.heliyon.2024.e30970

**Published:** 2024-05-10

**Authors:** Jiří Trousil, Joao Victor Cabral, Eleni Voukali, Jitka Nováčková, Ognen Pop-Georgievski, Tomáš Vacík, Pavel Studený, Hana Studenovska, Katerina Jirsova

**Affiliations:** a Institute of Macromolecular Chemistry, Czech Academy of Sciences, Prague, Czech Republic; b Institute of Biology and Medical Genetics, First Faculty of Medicine, Charles University and General University Hospital in Prague, Prague, Czech Republic; c Ophthalmology Department, Third Faculty of Medicine, Charles University and University Hospital Kralovske Vinohrady, Prague, Czech Republic

**Keywords:** Limbal epithelial stem cells, Biomaterial, Tissue sealant, Ocular tissue engineering, PDLLA

## Abstract

Ultrathin electrospun poly (l-lactide-*co*-dl-lactide) nanofibrous membranes coated with fibronectin were explored as scaffolds for the *ex vivo* cultivation of limbal epithelial cells (LECs) for the treatment of limbal stem cell deficiency. The developed scaffolds were compared with the “gold-standard” fibrin gel. The resulting membranes composed of nanofibers possessed a very low thickness of 4 μm and allowed very good optical transparency in the wet state. The fibronectin-coated nanofibrous scaffolds demonstrated LEC expansion and successful cultivation similar to that on fibrin gel. Unlike the regular cobblestone epithelial cell morphology on the fibrin gel, the nanofibrous scaffold presented a mostly irregular epithelial morphology with a shift to a mesenchymal phenotype, as confirmed by the upregulation of profibroblastic genes: *ACTA2* (*p* = 0.023), *FBLN1* (*p* < 0.001), and *THY1* (*p* < 0.001). Both culture conditions revealed comparable expression of stem cell markers, including *KLF4, ΔNp63α and ABCG2*, emphasizing the promise of polylactide-based nanofibrous membranes for further investigations.

## Graphical abstract

We describe ultrathin electrospun poly (l-lactide-*co*-dl-lactide) nanofibrous membranes as a scaffold for the *ex vivo* cultivation of limbal epithelial cells. We show that the nanofibrous scaffolds possess a very low thickness of 4 μm, allow for a very good optical transparency in the wet state, and demonstrate limbal epithelial cells expansion and successful cultivation that reveals high expression of stem cell markers, including ΔNp63α, OCT4, and NGFR.

**Figure:**
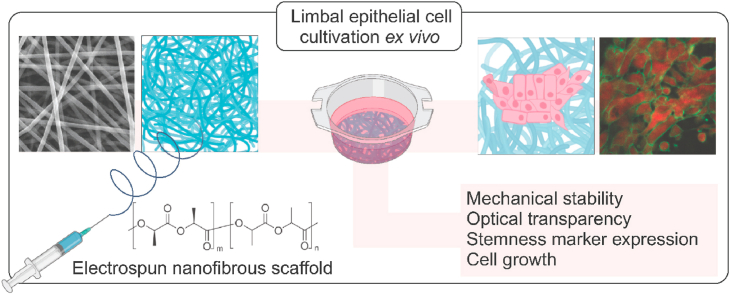

## 1 Introduction

Limbal epithelial stem cells (LESCs) are unipotent stem cells residing in a stem cell niche within the limbus [1]. This niche is a specialized compartment with unique properties in terms of physical, autocrine, paracrine, and multicellular homeostasis [2]. LESCs are nested deep in the basal epithelial layer of the limbus and are responsible for the lifelong regeneration of the corneal epithelium. Limbal damage, whether partial or total, unilateral or bilateral, resulting from acquired or genetic conditions such as ocular burns, Stevens-Johnson syndrome, or aniridia can lead to a reduction or absence of LESCs, thereby causing dysfunction in homeostasis [3,4]. This condition, limbal stem cell deficiency (LSCD), disrupts the barrier between the cornea and conjunctiva, leading to the outgrowth of conjunctival cells over the corneal surface (conjunctivalization), vascularization, chronic inflammation, photophobia, recurrent pain, and, finally, decreased or lost vision [5,6]. *Ex vivo* cultivated limbal epithelium transplantation or simple limbal epithelial transplantation are the most efficient procedure for the treatment of unilateral LSCD. In the case of patients suffering from bilateral total LSCD, however, limbal tissue is not available for such interventions. In this case, the transplantation of oral mucosa epithelial cells (OMECs) is one of the most efficient approaches [6]. A rejection of allografts limits the long-term prognosis in cases of allogenic limbal transplantation, thereby requiring systemic immunosuppression [7].

Current research aims to develop appropriate scaffolds and cultivation strategies to replicate the biological niche *in vitro* with a reduced number of shortcomings as well as immune responses [3,6]. Fibrin gel represents a widely used biomaterial for LEC *ex vivo* cultivation [8,9]. It is a biodegradable biopolymer formed from fibrinogen and thrombin that has emerged as a promising material in regenerative medicine and tissue engineering [[10], [11], [12]]. Fibrin gel promotes cell expansion, maintains LESC stemness, and allows for transfer to the patient's eye [13,14]. Both weak mechanical properties and shrinkage of the hydrogel are potential obstacles to be addressed [15,16].

In the case of OMECs, human amniotic membrane (HAM) is a widely used substrate for OMEC cultivation [[17], [18], [19]]; using HAM, successful LEC cultivation was previously described [20,21]. Pioneered by Nakamura and coworkers [17], OMEC cultivation using HAM minimizes the risk of graft rejection and possesses the advantage of making it possible to repeat the procedure. Neoangiogenesis and corneal vascularization following the transplantation procedure, however, complicate this approach [6]. HAM is, however, an allogenic material; while it has been widely studied in the field of ocular surface regeneration [21,22], it bears a potential risk of infectious disease transmission and contamination, and its clinical use can be potentially complicated by limited tissue availability and biological variability [23]. The use of biomaterials and synthetic scaffolds offers attractive opportunities to address clinical shortcomings. Recently, many alternative materials have been used for culturing LESCs. For example, Haagdorens and coworkers investigated the performance of surface-modified crosslinked collagen scaffolds [24]. They studied various collagen-derived materials, namely, 4-(4,6-dimethoxy-1,3,5-triazin-2-yl)-4-methyl-morpholinium chloride-crosslinked collagen-like peptide hydrogel, carbodiimide/N-hydroxysuccinimide-crosslinked collagen-like peptide hydrogel, and N-(3-dimethylaminopropyl)-N′-ethylcarbodiimide hydrochloride/N-hydroxysuccinimide-crosslinked plant-derived recombinant human collagen type I hydrogel, with regard to immortalized human corneal epithelial cell and primary human limbal epithelial cell cultivation. Upon confluence, primary LECs revealed high expression of the stem cell marker ΔNp63, keratin (KRT) 14, adhesion markers integrin-β4 and E-cadherin, and extracellular matrix proteins laminin-α1 and collagen type IV [24]. In the same endeavor, fibrous polymeric membranes have been used as scaffolds for the cultivation of ocular tissue cells [25]. High permeability of membranes was found to be the main factor allowing successful eye graft functioning [26,27].

Electrospun membranes prepared from biodegradable polymers can merge the advantages of high porosity, large pore size and even low thickness. For example, electrospun membranes were fabricated from poly (l-lactide) [28,29] or different lactide copolymers such as poly (l-lactide-*co*-d,l-lactide) [30,31], poly (lactide-*co*-glycolide) [32,33], and poly (l-lactide-*co*-caprolactone) [34]. Sanie-Jahromi and coworkers addressed polycaprolactone-based fibrous scaffolds prepared by electrospinning. The fabricated scaffolds led to good cell adherence, epithelial cell proliferation, and epithelial regeneration in an animal model [35]. Similarly, the Mijović group studied biocompatible polylactic acid modified by silk fibroin and gelatine. Notably, the fabricated electrospun scaffolds revealed higher adherence of LESCs; cell growth, proliferation, and corneal epithelial differentiation were confirmed by the expression of specific markers [36].

In this work, ultrathin electrospun poly (l-lactide-*co*-dl-lactide) (PDLLA) nanofibrous membrane scaffolds were studied as substrates suitable for LEC cultivation. The prepared membranes were comprehensively characterized to unravel their morphological, chemical, and optical transparency properties. Subsequently, the developed PDLLA membranes coated with fibronectin were compared with fibrin gel previously described as a “golden” substrate for LESC cultivation [8,9]. A detailed biorelevant study of a nanofibrous membrane was addressed, and the expression of specific genes and the stemness, proliferation, and differentiation were analysed. The developed concept of merging electrospun PDLLA nanofibrous membranes with fibronectin contributes to the field of LESC cultivation, thus possibly paving the way towards safe clinical trials employing the cultivation of eye tissue cells.

## 2 Materials and methods

### 2.1 Nanofibrous membrane fabrication

PDLLA (dl-LA/l-LA monomer ratio 10/90, *M*_w_ 868 270 g/mol, PDI 2.3) used for this study was prepared by a ring-opening polymerization of l-lactide and dl-lactide (Sigma‒Aldrich, Prague, Czech Republic) using tin (II)octanoate as a catalyst as previously described [37].

Nanofibrous membranes were prepared by electrospinning PDLLA from pyridine (9 wt%). Smooth bead-free fibres were generated at a voltage of 7 kV, 10 cm distant from the surface of the grounded collector, and a flow rate of 250 μL/h using a 20 G single needle nozzle. Nanofibers were collected on silicon wafers 18 × 18 mm (Siegert Wafer GmbH, Aachen, Germany) modified with sodium alginate solution (30 mg/mL in water) to allow complete detachment of the membrane from the wafer surface while dipped in water. After preparation, the membranes were dried for 24 h at 75 °C. Membranes were floated on a water surface, transferred to a PTFE foil surface, and fixed by silicon glue to the body of the cultivation insert (Corning Inc., Kennenburg, ME, USA) after removal of the original membrane.

Before limbal explant seeding (see below), the fabricated scaffolds were sterilized by soaking in 70 % ethanol (#E03801, Penta, Prague, Czech Republic) and washed three times with sterile PBS. Thereafter, in a 12-well plate, human fibronectin solution (600 μL, 10 μg/mL in PBS, #10838039001, Sigma‒Aldrich) was pipetted into each insert carrying the scaffolds. The plate was incubated for 1 h at 37 °C; subsequently, the inserts were washed three times with sterile PBS and then left to dry upside down in a laminar flow cabinet for 30 min. The nanofibrous membrane scaffold rehydration was carried out by pipetting 600 μL and 600 μL of DMEM/F12 medium supplemented with 1 % of an antibiotic-antimycotic solution (AA, #15240-062, Thermo Fisher Scientific, Waltham, MA, USA) inside and outside each insert, respectively. Rehydration was carried out for 30 min at 37 °C.

### 2.2 Nanofibrous membrane characterization

The membrane thickness was measured from the highest step of the groove made in the sample by a KLA-Tencor P-10 Surface Profiler (KLA-Tencor, Milpitas, CA, USA) after platinum sputter coating. The membrane thickness was defined as the average of the ten highest peaks in the profile.

The fibre quality and the fibre thickness were analysed using scanning electron microscopy (VEGA Plus, Tescan, Brno, Czech Republic) after sputter coating with platinum before visualization. The fibre width was assessed using VEGA Plus software (Tescan) at 20 randomly chosen locations in three images. As previously described [38], the porosity of the nanofibrous membrane was calculated using the experimentally determined areal density, membrane thickness, and fibre material density of 1.248 g/cm^3^ [39].

The areal density of the known area of membranes was evaluated by weighing (METTLER TOLEDO MX5 microbalance, Columbus, OH, USA).

### 2.3 X-ray photoelectron spectroscopy

Successful coating of the nanofibrous membrane with fibronectin was indicated by X-ray photoelectron spectroscopy (XPS) measurements. It was carried out with a K-Alpha^+^ spectrometer (Thermo Fisher Scientific, East Grinstead, UK). The samples were analysed using a microfocused, monochromated Al Kα X-ray source at an angle of incidence of 30° (measured from the surface) and an emission angle normal to the surface. The kinetic energy of the electrons was measured using a 180° hemispherical energy analyser operated in the constant analyser energy mode (CAE) at 200 eV and 50 eV pass energy for the survey and high-resolution spectra, respectively. A dual-charge compensation system was utilized to limit the surface charge build-up. High-resolution C 1s, N 1s, and O 1s core level and survey spectra were measured at a minimum of 9 points over the surfaces of pristine nonmodified fibrous PDLLA membranes, fibronectin films formed on gold surfaces and fibronectin-modified PDLLA membranes. Spectral resolutions of 0.1 and 1.0 eV were used for the high-resolution and survey spectra, respectively. All reported XPS spectra are referenced to the C 1s peak of hydrocarbons at 285.0 eV. Data acquisition and processing were performed using Thermo Advantage software (Thermo Fisher Scientific). The XPS spectra were fitted with Voigt profiles obtained by convolving Lorentzian and Gaussian functions. The analyser transmission function, Scofield sensitivity factors, and effective attenuation lengths (EALs) for photoelectrons were applied for quantification. EALs were calculated using the standard TPP-2M formalism. The BE scale was controlled by the well-known position of the photoelectron C–C and C–H, C–O and C (=O)–O C 1s peaks of polyethylene terephthalate and Cu 2p, Ag 3d, and Au 4f peaks of metallic Cu, Ag, and Au, respectively. The BE uncertainty of the reported measurements and analysis is in the range of ±0.1 eV.

### 2.4 Fibrin gel preparation

Fibrin gels were prepared using the fibrin-based sealant Tisseel Lyo (Baxter Health care Ltd., Norfolk, UK). According to the manufacturer's instructions, the fibrinogen solution was prepared by dissolving the lyophilized pellet in an aprotinin solution. The thrombin solution was prepared by dissolving lyophilized human thrombin in calcium chloride solution. Following rehydration, both solutions were further diluted with sterile PBS. The final concentrations of fibrinogen and aprotinin were 10 mg/mL and 333 000 IU/mL, respectively. The final concentrations of thrombin and calcium chloride were 10 IU/mL and 0.8 μmol/mL, respectively.

Fibrin gel preparation was carried out in a 12-well plate (Corning Inc., Corning, NY, USA). Prewarmed (37 °C) fibrinogen solution (300 μL) was added to each well, followed by mixing with prewarmed (37 °C) thrombin solution (300 μL). The solutions were mixed using a pipette tip attached to a pipette.

Note that the resulting fibrin layer thickness was calculated to be 1.58 mm in a 12-well plate; for the light transmittance measurement (see below), the same fibrin gel thickness was used.

### 2.5 Optical properties

The optical transparency of the nanofibrous membrane and fibrin gel was measured as a light transmittance (%T) in a visible range of wavelengths 400–700 nm using a Perkin-Elmer Lambda 35 UV‒VIS spectrometer (Waltham, MA, USA). Wet nanofibrous membranes were prepared by pretreatment with 70 % ethanol (1 min) and dipping in distilled water for 2 h. The light transmittance of both dry and wet membranes fixed to an aluminium frame was measured between two coverslips. Fibrin gel was prepared in a commercial polystyrene well fixed to the surface of a glass coverslip; the wells were 11 mm in diameter (equivalent to 12-well plates used for the *ex vivo* cultivations). Air (one or two coverslips) was used as a reference; a coverslip with no sample was used as a control.

Visual optical transparency of both culture scaffolds was assessed over a printed text and photo documented.

### 2.6 Donor corneal tissues

The study adhered to the tenets set out in the Declaration of Helsinki; donor tissue procurement met all Czech legal requirements, including the absence of the donor in the national register of persons opposed to postmortem withdrawal of tissues and organs. Based on the Czech legislation on specific health services (Law Act No. 372/2011 Coll.), informed consent is not required if the presented data are anonymized in the form.

Corneoscleral rims were obtained from seven cadaveric donor corneas within 24 h after death. The mean age of the donors was 57 years (ranging from 41 to 75), with 6 males and 1 female. The tissues were stored in Eusol-C preservation medium (#CSM 001-00, Alchimia, Padova, Italy) for an average of 10.6 days before seeding. All rims were used for the cultivation on fibrin gel and PDLLA scaffolds.

### 2.7 Preparation and seeding of limbal explants

Donor corneoscleral rims were washed three times in Dulbecco's modified Eagle's medium (DMEM)/F12 (#11320033, Thermo Fisher Scientific) containing AA. The AA supplementation was 1, 10, and 1 % in the first (3 min), second (3 min), and third (3 min) washing steps, respectively. Subsequently, each corneoscleral rim was cut into 12 pcs (ca. 2 × 3 mm in size) and washed (3 × 3 min) with DMEM/F12 medium supplemented with 1 % AA.

Thereafter, 6 pcs of the explants were placed in the wells containing the fibrin gels, whereas the other 6 pcs were placed in the wells containing the nanofibrous fibronectin-coated membranes. The explant was oriented so that the surface of the epithelium was in contact with the culture surface (i.e., fibrin gel or nanofibrous membrane). Cells were cultured in a complex medium (COM) consisting of DMEM/F12, 10 % human serum (#HU.SE.0500, Bio&Sell, Nuremberg, Germany), 1 % AA, 10 ng/mL recombinant EGF (#PH60311L, Thermo Fisher Scientific), 0.5 % insulin-transferrin-selenium supplement (#41400045, Thermo Fisher Scientific), 5 μg/mL hydrocortisone (#H0888, Sigma‒Aldrich), 10 μg/mL adenine hydrochloride (#A9795, Sigma‒Aldrich), and 10 ng/mL cholera toxin (#C8052, Sigma‒Aldrich). Tranexamic acid (#PHR1812, Sigma‒Aldrich) was used as a COM supplement at 160 μg/mL in the case of fibrin gels only. The plates were incubated at 37 °C in a humidified atmosphere containing 10 % CO_2_. Initially (0 days post seeding, dps), fibrin gel- and nanofibrous membrane-cultured explants were covered with 100 μL and 1100 μL of COM, respectively, to avoid the explant detachment of the surface. The next day (1 dps), the COM volume was increased to 300 μL and 1200 μL for fibrin- and membrane-cultured explants, respectively, considering that the explant was already firmly attached to the fibrin gel or nanofibrous membrane surface. The medium was changed three times a week until the cells reached approximately 90–100 % confluence. Throughout the cultivation, the explants were monitored for cell morphology and growth, confluence, and the presence of fibroblast-like cells using an inverted microscope (Olympus CKX41, Olympus, Tokyo, Japan) coupled with an EOS 250D camera (Olympus); images were acquired using QuickPhoto Camera software (Promicra, Prague, Czech Republic). To estimate confluence, the amount of space covered by the expanding cells was compared with the unoccupied space by the naked eye.

Haematoxylin-eosin (H&E) staining was applied to cell cultures grown on PDLLA nanofibrous membranes. The membranes were fixed with prechilled 4 % paraformaldehyde (#76240, Fluka Chemical) for 15 min, washed in PBS for 5 min, and then stained with Haematoxylin Gill (#GHS132, Sigma‒Aldrich) for 2 min, followed by three washes with tap water. Filtered 2 % eosin (#17372-87-1, Merck) was added for 1 min, and the membrane was rinsed twice with distilled water. Stained membranes were observed using an inverted microscope (Olympus CKX41, Olympus, Tokyo, Japan) coupled with an EOS 250D camera (Olympus). Images were acquired using QuickPhoto Camera software (Promicra, Prague, Czech Republic).

### 2.8 Cell lysis and RT‒qPCR

Cells from seven donors (cultured on both fibrin gels and PDLLA scaffolds) were used, respectively. Once the expanding cells reached 90–100 % confluence, sterile forceps were used to carefully remove the explants while keeping the cell sheet attached to the scaffold. Fibrin gel-based cultures were harvested enzymatically. The gels were washed twice with 0.5 mL of a sterile dispase II solution (1 U/mL in PBS, #17105-041, Gibco, Thermo Fisher Scientific) at room temperature (RT). Next, the gels were detached and disintegrated using sterile forceps and scissors; 1 mL of the dispase II solution was added to each well, and the plate was incubated for 30 min at 37 °C. The gel digestion and cell sheet release were occasionally controlled by mixing each well content using a pipette. The released cell sheets related to a representative experimental group were pooled and centrifuged (250×*g*, 10 min, 4 °C). The obtained pellet was incubated with TrypLE™ Express Enzyme reagent (2 mL per pooled sample, #12604013) for 25 min at 37 °C; mixing using a pipette was applied occasionally. The cells were obtained by centrifugation (250×*g*, 10 min, 4 °C), and the resulting cell pellet was directly lysed using 350 μL of RLT Plus buffer (#1053393, Qiagen, Hilden, Germany).

The nanofibrous membrane-based cultures were directly lysed using the same amount of RLT Plus buffer (Qiagen). Using one portion of the lysis buffer, 3–6 nanofibrous membrane culture samples (inserts) were pooled for RT‒qPCR analysis. The lysates were stored at −80 °C before RT‒qPCR analysis. RNA extraction was carried out using an RNeasy Micro Kit (#74004, Qiagen) according to the manufacturer's instructions. The obtained RNA was diluted in RNase-free water. RNA quantification and quality were assessed using the 260/280 nm and 260/230 nm ratios. For this, an Eppendorf BioPhotometer spectrophotometer (#6131, Eppendorf, Hamburg, Germany) was used. The RNA quality was further assessed by agarose gel electrophoresis. The RNA concentrations of each sample were measured and diluted accordingly to the same concentration (25 ng/μL). cDNA synthesis was carried out using an iScript cDNA synthesis kit (#1708890, Bio-Rad, Hercules, CA, USA).

RT‒qPCR was carried out in a Hard Shell 96-well PCR plate (#HSR9901, Bio-Rad) using a SsoAdvanced Universal SYBR Green Supermix RT‒qPCR Kit (#1725270, Bio-Rad); 7 samples were used for each marker and each group in 3 technical replicates. The results were evaluated using a Bio-Rad detection system (CFX Connect Real-Time PCR Detection System, Bio-Rad). Controls without templates were included for each primer pair to check for any contaminants. As another quality control measure, the melting (dissociation) curves of qPCRs were monitored to ensure that there was only a single RT‒qPCR product and no primer dimers. The specifications of all primers used are stated in Table 1.

**Table 1:** Primers used for the RT-qPCR analysis.

Gene name and symbol	Orward primer	Reverse primer	Size (bp)
Housekeeping genes			
Hypoxanthine phosphoribosyltransferase 1, *HPRT1*	TCTTTGCTGACCTGCTGGATTAC	GTCTGCATTGTTTTGCCAGTGTC	214
Ribosomal protein L32, *RPL32*	CTCAGACCCCTTGTGAAGCC	TTGCTTCCATAACCAATGTTGG	179
Progenitor and putative stem cell markers			
POU class 5 homeobox 1, *OCT4*	AGAAGTGGGTGGAGGAAGCTG	CCAGGTTGCCTCTCACTCG	123
SRY-box transcription Factor 2, *SOX2*	GCTAGTCTCCAAGCGACGAAA	GCCTCTCCTTGAAAAATATTGGC	137
Tumour protein p63, *ΔNp63α*	TATCCGCATGCAGGACTCG	GAGCCAGAAGAAAGGACAGCAG	127
ATP binding cassette subfamily G member 2 (Junior blood group), *ABCG2*	GAGCCTACAACTGGCTTAGACTCAA	TGATTGTTCGTCCCTGCTTAGAC	85
ATP-binding cassette subfamily B member 5, *ABCB5*	CAGCAAGGGAAGCAAATGC	GGGTTTCGAACTAAGGCACG	139
Nerve growth factor receptor, *NGFR*	CGTATTCCGACGAGGCCA	ACCGTGTAATCCAACGGCC	138
CCAAT enhancer binding protein delta, *CEBPD*	GAGAACGAGAAGCTGCACCAG	TGAGGTATGGGTCGTTGCTG	170
Leucine-rich repeats and immunoglobulin-like domains 1, *LRIG1*	CCGTGGCTAATTGGCAGG	TGTCCTTGCCCACCATAGC	178
Proliferation markers			
Kruppel-like Factor 4, *KLF4*	CCACACTTGTGATTACGCGG	GAATTTCCATCCACAGCCGT	127
Marker of proliferation Ki-67, *MKI67*	CTTTGGGTGCGACTTGACG	GTCGACCCCGCTCCTTTT	19
Insulin-like growth factor binding protein 5, *IGFBP5*	AGCTACCGCGAGCAAGTCAA	TCGGAGATGCGGGTGTGT	125
Differentiation markers			
Keratin 3, *KRT3*	GGATGTGGACAGTGCCTATATGAA	AGCACCACAGATGTGTCACTGAT	144
Keratin 7, *KRT7*	ATGGAGTGGGAGCCGTGAA	AGCCTTCAGGAGCCCAGG	146
Keratin 8, *KRT8*	CGAGATCGCCACCTACAGGA	AGCTCAGACCACCTGCATAGC	116
Keratin 12, *KRT12*	GGAGATCGAGCTACAGTCCCA	TCCAGGTTGCTGATGAGCTG	120
Keratin 14, *KRT14*	TCTCCTCTGGATCGCAGTCA	GCCTCAGTTCTTGGTGCGA	131
Keratin 15, *KRT15*	AGGGCCTGAATGAGGAGCTAG	CCTCATCTCTGCCAGCACAC	146
Fibroblast markers			
Actin alpha 2, smooth muscle, *ACTA2*	CTTTGCTGGGGACGATGC	TCCCATTCCCACCATCACC	85
CD34 molecule, *CD34*	GGCATCTGCCTGGAGCAA	CACCTCAGACTGGGCAAGGA	153
Thy-1 cell surface antigen, *THY1*	TCCCCACCCATCTCCTCC	CGAGGTGTTCTGAGCCAGC	90
Visual system homeobox 2, *VSX2*	AAGAAACGGAAGAAGCGGC	TGGGTAGTGGGCTTCGTTG	91
Fibulin 1, *FBLN1*	CTGCGAATGCAAGACGGG	CAGCGTGTTCTCGCACTTGT	115

### 2.9 Cell imaging and indirect immunofluorescence

Two samples were used for cell visualization and immunostaining (both after cultivation on fibrin and PDLLA). Cells were visualized using eBioscience™ Calcein Violet 450 a.m. Viability Dye (#65-0854-39, Thermo Fisher Scientific) and CellMask™ Deep Red Plasma Membrane Stain (#C10046, Thermo Fisher Scientific) according to the manufacturer's instructions. Then, the cells were washed with PBS (3 × 10 min) and immediately visualized. The PDLLA nanofibrous membrane inserts were placed onto a glass-bottom dish (MatTek Corp., Ashland, MA, USA); PBS was added (200 μL) inside the insert to protect the sample from drying. Fibrin gels were released from the cultivation plate with a spatula and placed onto a glass-bottom dish (MatTek Corp.) upside down. An Olympus IX83 confocal laser scanning microscope (Olympus Czech Group Ltd., Prague, Czechia) equipped with a 60× oil objective (PLAPON 60XO, NA 1.42) was used for cell imaging at channels Ch 1 (excitation 405, emission 425–475) and Ch 2 (excitation 635, emission 650–750).

The presence of selected markers was visualized using immunofluorescence staining. For this purpose, explant cultivation was followed by washing (3 × 5 min) in PBS at RT and fixation with prechilled 4 % paraformaldehyde (15 min). Next, the samples were washed with PBS (3 × 10 min), permeabilized with Triton X-100 (0.3 %, PBS) for 10 min at RT, washed with PBS (3 × 10 min), and blocked with a solution (1 h, RT) containing 2.5 % goat serum (#C07SAZ, Bio-Rad, Hercules, CA, USA), 2.5 % BSA (#A9647, Sigma‒Aldrich), and 0.3 % Triton X-100 in PBS. Incubation with anti-p63α rabbit polyclonal antibody (#4892S, Cell Signalling, Danvers, MA, USA) and anti-keratin 7 mouse monoclonal antibody (#OV-TL-12/30, Zeta Corporation, Arcadia, CA, USA) at a dilution of 1/250 was carried out at 4 °C overnight. Next, the samples were washed with PBS (3 × 10 min) and incubated with secondary antibodies, namely, Alexa Fluor 488-conjugated goat anti-rabbit IgG (1/250, #A-11008, Thermo Fisher Scientific) and Alexa Fluor 594-conjugated goat anti-mouse IgG (1/250, #A-11032, Thermo Fisher Scientific), at 4 °C overnight. Next, the samples were washed, counterstained with 4′,6-diamidino-2-phenylindole (DAPI) and imaged as described above: Ch 1 (excitation 405 nm, emission 425–475), Ch 2 (excitation 488 nm, emission 500–530 nm), and Ch 3 (excitation 543 nm, emission 560–660 nm).

### 2.10 Data evaluation and statistical analysis

The statistical analyses of the qPCR results were conducted with R software [40] (version 4.2.3) using, among others, the packages ggplot2 [41] and pheatmap for visualization. Given their non-Gaussian distribution, the relative mRNA expression values were normalized using decadic logarithms. First, the expression data relative to ribosomal protein L32 (*RPL32*) were plotted in a heatmap generated using the pheatmap package. Next, the comparisons between PDLLA (referred to as PDLLA scaffolds 1–7) and their paired fibrin gel-based samples (referred to as fibrin gels 1–7) on gene expression changes were assessed using a paired *t*-test for each marker, following assessment of data normality with the Shapiro test. Differences were considered significant at *p* ≤ 0.05.

## 3 Results

### 3.1 Nanofibrous membrane preparation and characterization

**Fig. 1:**
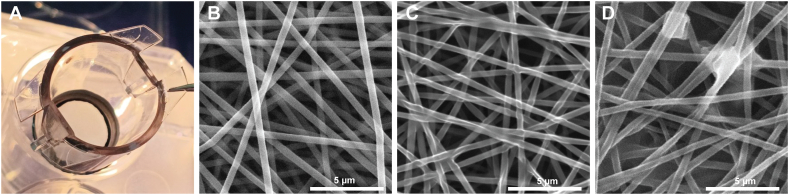
PDLLA nanofibrous membrane characteristics. (A) Gross view of the based nanofibrous membrane fixed to a cell culture insert for 12-well plates. (B) Structure of the neat PDLLA nanofibrous membrane as shown by SEM. (C) Neat PDLLA nanofibrous membrane sterilized by soaking in ethanol as described in the experimental section. (D) Nanofibrous membrane sterilized by soaking in ethanol and coated with fibronectin at 10 μg/mL as shown by SEM. Note the protein deposit foci evident secondary to the fibronectin coating.

The PDLLA electrospun membranes were prepared, and the resulting design of the cultivation cup/insert bearing the PDLLA-based nanofibrous membrane is depicted in Fig. 1A.

The thickness of the fabricated membrane measured by a profiler was found to be 4.1 μm, and the calculated porosity was 72 %. The thickness of bead-free fibres was 328 ± 27 nm (mean ± SD; *n* = 60); the structure of the mat can be seen in Fig. 1B. The areal density of nanofibrous membranes was found to be 143 ± 4 μg/cm^2^ (mean ± SD; *n* = 10), which is in good agreement with the previously reported minimum value of nanofibrous membranes suitable for surgical treatment, namely, 90 μg/cm^2^ [38].

**Fig. 2:**
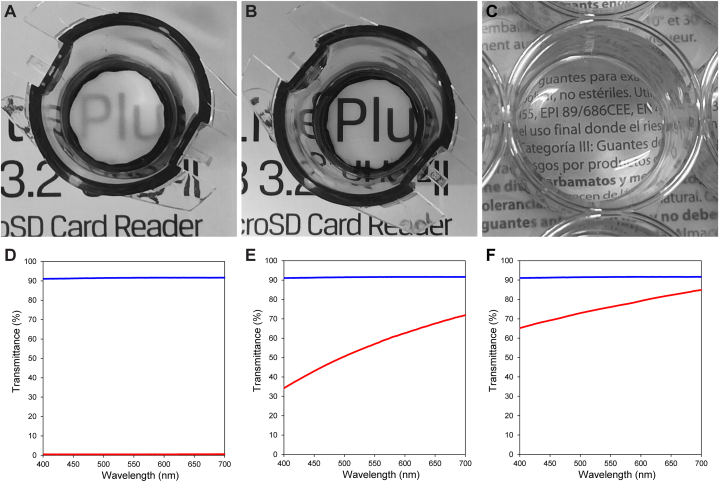
Optical transparency of the studied cell culture scaffolds. Gross view of the scaffolds and resulting optical transparency. (A) Dry nanofibrous PDLLA membrane reveals a partial opacity only. Both the wet PDLLA membrane (B) and fibrin gel (C) possess high transparency. PDLLA membranes are presented as 12-well plate inserts prepared in-house; fibrin gel is presented as a gel (600 μL) prepared in a 12-well plate. (D, E, F) Corresponding light transmittance was assessed. Transmittance spectra of the dry nanofibrous PDLLA membrane (D), wet nanofibrous PDLLA membrane (E), and fibrin gel (F). The transmittance of a glass coverslip (control) was also assessed (blue line).

Both the fibrin gel and PDLLA membranes were assessed for scaffold optical transparency measurement. Fig. 2 shows a photograph for visual assessment of the optical transparency of the dry PDLLA nanofibrous membrane (Fig. 2A), wet nanofibrous membrane (Fig. 2B), and fibrin gel (Fig. 2C). The dry membrane was opaque, and the text underneath could not be read through it. Both the wet PDLLA membrane and the fibrin gel revealed transparency; the text underneath was well visible. The transparency of these three samples was also assessed via UV‒VIS spectroscopy. The resulting transmittance spectra are depicted (Fig. 2D–F). Light transmittance values (%T, mean ± SD, *n* = 3) measured at 600 nm were (0.63 ± 0.17), (61.3 ± 1.4), (80.1 ± 1.2), and (91.6 ± 0.1) for the dry nanofibrous membrane, wet nanofibrous membrane, fibrin gel, and glass coverslip (control), respectively.

Fibronectin-coated PDLLA membranes (10 μg/mL) were further characterized by means of both SEM and XPS. SEM imaging of the fibronectin-coated membranes (Fig. 1D) revealed multifocal heterogeneous deposits of the protein; both the neat membranes (Fig. 1B) and membranes sterilized by soaking in 70 % ethanol and washed three times in sterile PBS (Fig. 1C) were free of any amorphous protein structures.

**Fig. 3:**
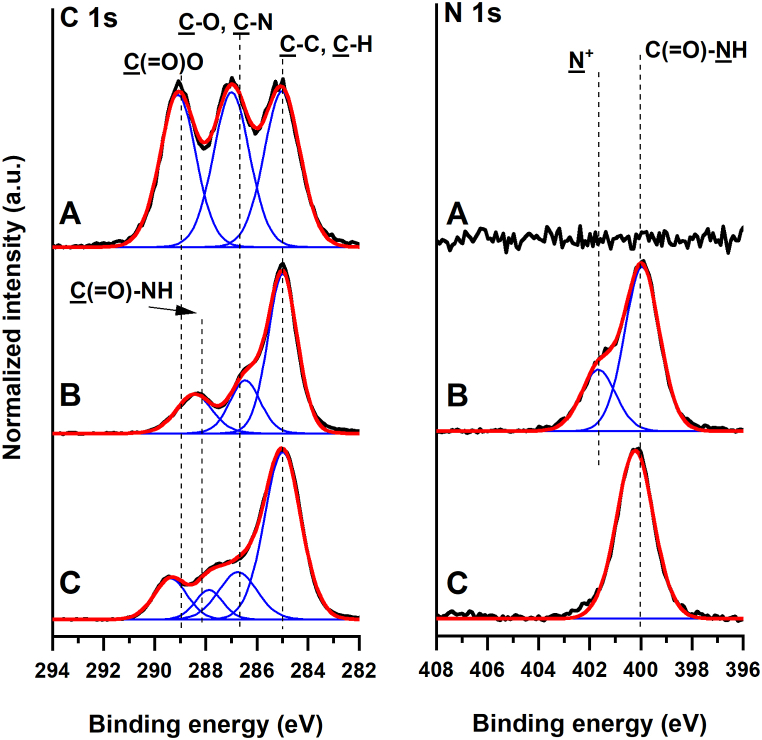
High-resolution XPS spectra taken in the C 1s and N 1s regions of the pristine nonmodified PDLLA nanofibrous membrane (A), reference fibronectin layer formed on the gold surface (B), and fibronectin-modified PDLLA nanofibrous membrane (C). Measured spectra are presented with black lines, while their corresponding fitted envelopes are presented with red lines. The individual contributions of different functional groups are represented with blue lines.

Successful coating by fibronectin through the surface of the nanofibrous membrane was also proven by XPS measurements. The covalent structure of the PDLLA nanofibrous scaffolds before and after the deposition of fibronectin was confirmed. The high-resolution C 1s XPS spectrum of the PDLLA fibrous scaffold (Fig. 3) could be deconvoluted into three C–C, C–O, and O–C]O peaks of equal intensity at 285.0, ∼287.0 and 289.1 eV arising from the lactide monomer units (Table 2), similar to the case of continuous PLLA films [42]. The XPS analysis showed a complete absence of nitrogen species on the surface of the pristine fibrous scaffold. The high-resolution C1s spectrum of the fibronectin layer formed on the reference gold surface could be deconvoluted to a C–C peak and C–O and C–N contributions at 285.0 and ∼286.5 eV, respectively. Notably, a characteristic amide C (=O)–NH peak was observed at ∼288.4 eV. The high-resolution N 1s XPS spectra of the fibronectin film were characterized with an amide C]O–NH peak at ∼400.0 eV and a contribution of charged nitrogen N^+^ moieties at 401.7 eV. The adsorption of fibronectin on the surface of the PDLLA scaffold led to significant changes in the measured C 1s XPS spectrum. A distinct amide contribution could be found upon deconvolution of the spectrum at ∼287.9 eV originating from the fibronectin present on the surface. Concomitantly, in the high-resolution N 1s XPS spectra, we observed a strong amide C]O–NH peak at ∼400.2 eV. Overall, the XPS analysis confirmed the successful modification of the PDLLA fibrous scaffold with fibronectin.

**Table 2:** Atomic % of chemical moieties present on the surfaces of non-modified PDLLA nanofibrous membrane, reference fibronectin layer formed on the gold surface, and fibronectin-modified PDLLA nanofibrous membrane as determined by XPS.

	Binding Energy	Neat PDLLA membrane	Fibronectin	Fibronectin-coated PDLLA membrane
	(eV)	(atomic %)		
C1s C–C, C–H	285.0	21.0 ± 0.7	35.7 ± 1.0	38.6 ± 1.0
C1s C–O, C–N	286.7 ± 0.3	19.9 ± 0.4	8.9 ± 0.4	11.5 ± 0.1
C1s C (=O)–NH	288.2 ± 0.2	-a	10.7 ± 0.3	5.1 ± 0.8
C1s C (=O)–O	289.1 ± 0.3	20.1 ± 0.1	–	8.5 ± 0.6
N1s NH–C (=O)	400.0 ± 0.1	–	5.8 ± 0.4	4.8 ± 0.4
N1s NH_3_^+^	401.6 ± 0.1	–	3.1 ± 0.4	–
O1s	532.0 ± 0.3	38.9 ± 0.3	35.9 ± 1.5	31.4 ± 0.7
Sum		100.0	100.0	100.0

a Below the detection limit of XPS, i.e. 0.1 atomic %.

### 3.2 LEC growth dynamics and morphological observations

**Fig. 4:**
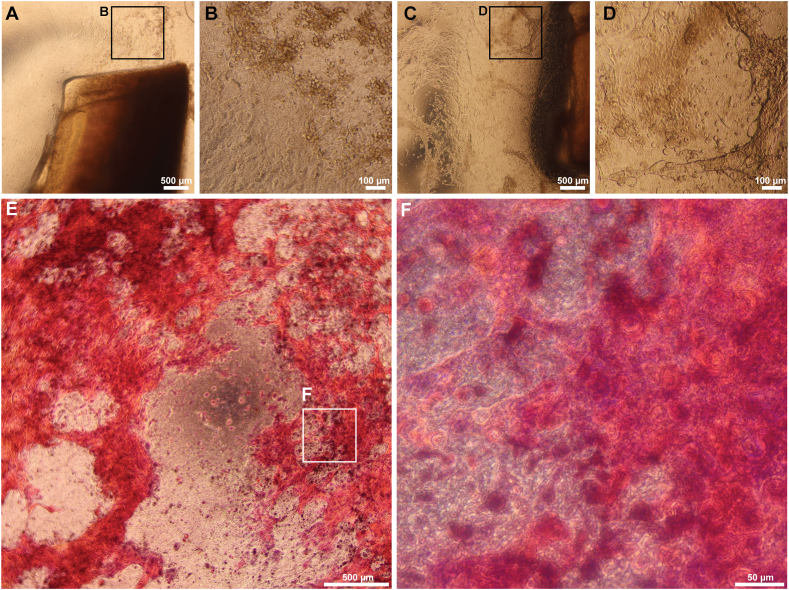
LEC cultivation on PDLLA nanofibrous membranes as observed by phase contrast light microscopy. A representative corneoscleral rim explant cultivated on a PDLLA membrane shows the beginning of LEC growth activity as well as the resulting morphology at 7 dps (A, B). The presence of both cobblestone and round cells was noted. At 14 dps, the growth was not regular, and both spindle-shaped cells and self-organizing structures were present at 14 dps (C, D). A representative self-organization of another LEC culture at 14 dps (E, F). H&E staining of a fixed PDLLA-based culture is shown.

To study LEC cultivation *ex vivo*, cell morphology and growth were assessed. Explants from seven donors were seeded onto the nanofibrous membrane substrate. The cell growth from explants cultured on PDLLA nanofibrous membranes started at 5–7 dps. Cell appearance was presented by both cobblestone morphology (Fig. 4A and B) and round cells. Furthermore, (7–10 dps), the cultures presented nonepithelial morphology and spindle-shaped cells that were characteristic of mesenchymal/fibroblasts. Cell migration across the substrate, multifocal growth, whorled patterns, and self-organizing structures occurred multifocally in most cases (Fig. 4C and D). The cultivated cells reached 90–100 % confluence at 16–21 dps; the cell growth patterns were confirmed using H&E staining of paraformaldehyde-fixed PDLLA membrane-based cultures (Fig. 4E and F). H&E also confirmed the multifocal presence of multicellular spheroids/organoids.

**Fig. 5:**
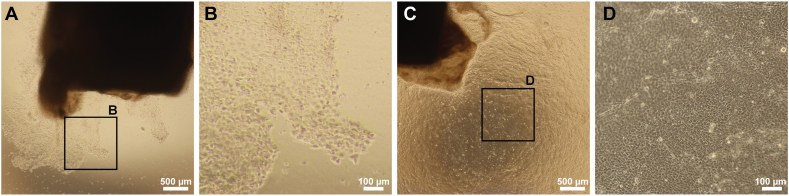
LEC cultivation on fibrin gel scaffold as observed in phase contrast of light microscopy. A representative corneoscleral rim explant on fibrin gel and observed LEC growth at 3 dps. The cells show uniform size and epithelial morphology (A, B). The same LEC culture reached ∼90 % confluence at 9 dps; a regularly organized monolayer possessing well-defined cobblestone cell morphology is shown (C, D).

For the fibrin gel culture study, the same experimental setup was followed; explants from seven donors were used. At 3–5 dps, the beginning of epithelial cell activity was observed among the seeded explants (Fig. 5A and B); the expanding cells revealed a typical epithelial cuboidal morphology. At 7 dps, the maximum percent growth observed was ∼70 %. Note that cell growth was faster in the case of fibrin gels; in contrast to the PDLLA nanofibrous substrate cultivations, the expanding cells reached 90–100 % confluence at 9–14 dps (Fig. 4C and D). Spindle cells were observed at the end of cultivation in 2 of the 10 donor tissues; however, their presence was negligible.

### 3.3 Cell fluorescence imaging

**Fig. 6:**
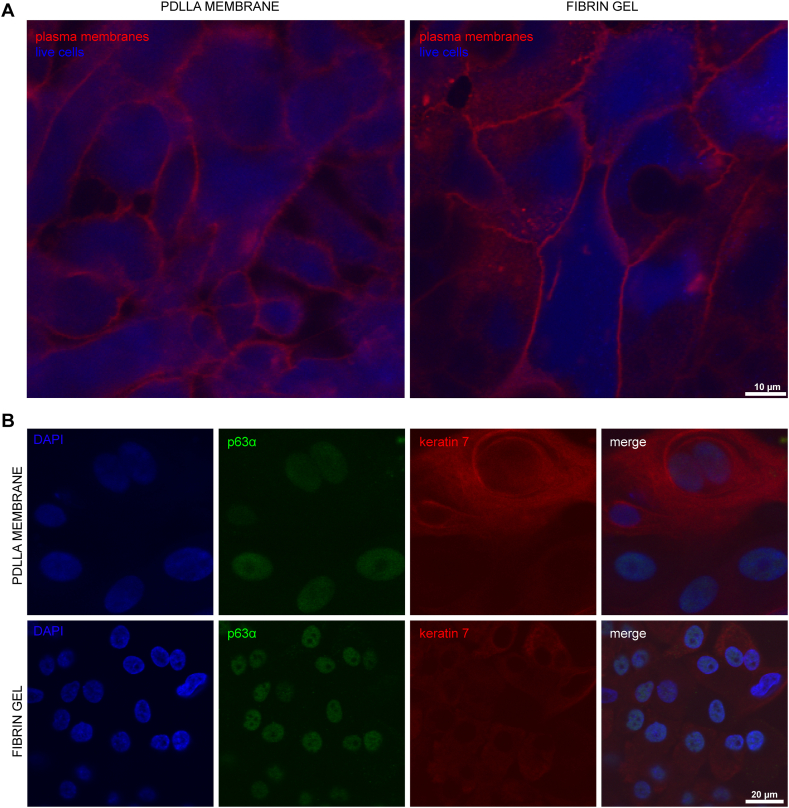
Cell appearance as found by live cell and immunofluorescence imaging. (A) Live cell staining (CytoCalcein Violet 450, blue). Plasma membranes were visualized using CellMask™ Plasma Membrane Deep Red staining (red). (B) Immunocytochemistry for both stemness marker p63α (green) and epithelial cell marker keratin 7 (red). Cell nuclei were counterstained with DAPI (blue). Note the smaller nuclei and cells in fibrin gel culture compared to PDLLA membrane culture.

In addition to phase contrast morphology and H&E staining, detailed staining and cell phenotype characterization were assessed. Visualization was performed by live cell (CytoCalcein Violet 450) and plasma membrane staining (CellMask™ Plasma Membrane Deep Red). Imaging revealed communicating and viable cells (Fig. 6A, blue) in both PDLLA scaffold and fibrin gel cultures. Plasma membrane staining revealed a culture of expanding cells, in line with the growth characteristics presented above (cf. Figs. 4 and 5).

Qualitative immunofluorescence imaging showed the presence of both the stemness marker p63α and one of the major structural proteins of epithelia, keratin 7, in cell nuclei and cytoplasm, respectively. Both antigens were present in both culture conditions, namely, PDLLA scaffold and fibrin gel cultivation (Fig. 6B).

### 3.4 Gene expression in cultured cells

**Fig. 7:**
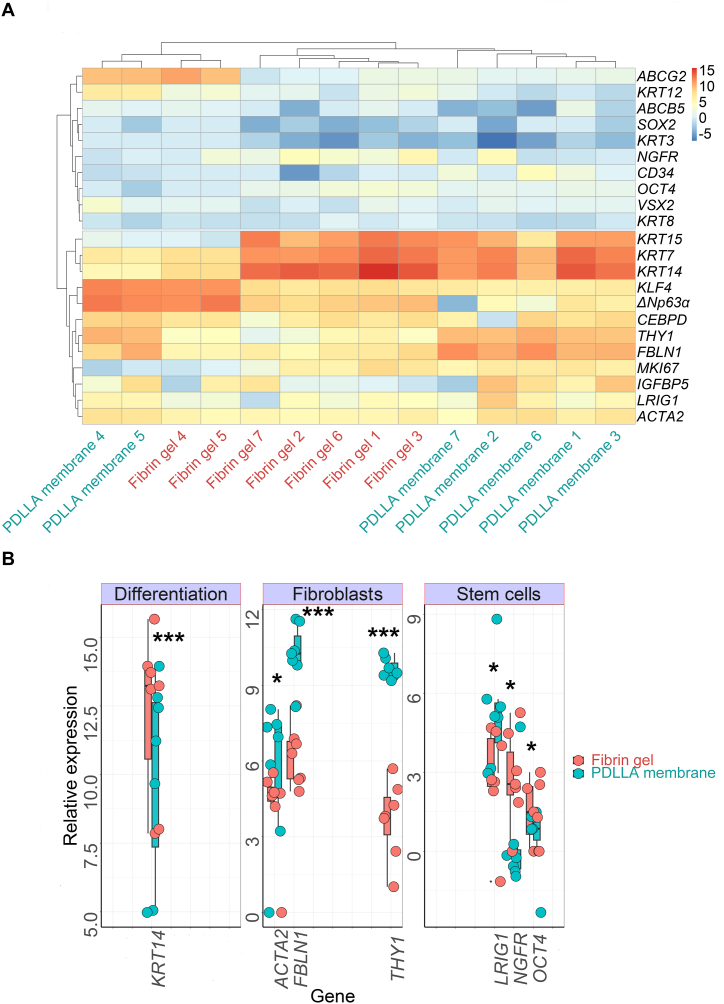
Gene expression of stem cell, proliferation, differentiation, and fibroblast markers assessed by real-time quantitative polymerase chain reaction in paired explant cultivation using the nanofibrous scaffold (PDLLA) or fibrin gels. (A) Overview of the relative mRNA expression (logarithmic) values of the tested genes (rows) represented as a heatmap. Seven nanofibrous scaffold samples are referred to as PDLLA scaffolds 1–7 and seven paired fibrin gel samples as fibrin gels 1–7. The bar on the right scales the higher expressions indicated in red and the lower in blue. (B) The boxplots show the gene expression of selected markers with statistically significant differences between the two experimental groups. Asterisks indicate the level of statistical significance as of *p* < 0.05 (*) and *p* < 0.001 (***).

Because the above-described morphological imaging indicated both stem cell and epithelial differentiation phenotypes, we further assessed the gene expression of stem cell, differentiation, proliferation, and mesenchymal cell/fibroblast markers in cells cultured on both the PDLLA membranes and fibrin gels. After bidirectional hierarchical clustering of the gene expression values, the samples were mostly grouped based on the cultivation method (substrate) used for cultivation (Fig. 7A). Furthermore, we observed that the donor had an impact on the sample grouping. A subset of donors exhibited elevated levels of *KLF4*, *ΔNp63α*, and *ABCG2* and lower levels of *KRT14*, *KRT15,* and *KRT7.* Overall, the genes were classified into 2 main clusters of rows, those that were highly expressed in most of the analysed samples and those with low expression levels. Most of the markers showed comparable expression under both cultivation conditions (*p* > 0.05, data not shown). Nevertheless, some progenitor and putative stem cell genes were significantly upregulated in fibrin gel-compared to PDLLA-based cultures. These genes included NGFR (paired *t*-test; t = 3.4353, df = 6, *p* = 0.014), OCT4 (paired *t*-test; t = 3.1722, df = 6, *p* = 0.019), and *KRT14* (paired *t*-test; t = 6.2584, df = 6, *p* < 0.001). In contrast, explant cultivation using the PDLLA nanofibrous scaffold led to a statistically significant upregulation of LRIG1 (paired *t*-test; t = −3.1025, df = 6, *p* = 0.021) and the mesenchymal cell/fibroblast markers *ACTA2* (paired *t*-test; t = −3.0448, df = 6, *p* = 0.023), *FBLN1* (paired *t*-test; t = −7.1639, df = 6, *p* < 0.001), and *THY1* (paired *t*-test; t = −9.9725, df = 6, *p* < 0.001) (Fig. 7B).

## 4 Discussion

The use of biomaterials and polymeric scaffolds offers attractive opportunities to address the clinical shortcomings related to LEC cultivation *ex vivo*. Therefore, we aimed to prepare ultrathin nanofibrous PDLLA membranes with favourable properties, including transparency, porosity, and membrane thickness, and compare their performance with that of fibrin gel, which is a standard substrate for LEC cultivation [8,9] and has emerged as a promising material in regenerative medicine and tissue engineering [[10], [11], [12]]. Although fibrin gel substrate has served as a gold standard for LEC cultivation, there are numerous obstacles, such as the weak mechanical properties of fibrin gels and shrinkage of the hydrogel, that need to be addressed for widespread adoption in tissue engineering [15,16]. Additionally, degradation of fibrin gel (up to one month [43]) is faster compared to the degradation of PDLLA nanofibrous scaffolds. The fabricated fibronectin-coated PDLLA nanofibrous membranes, however, could potentially allow mechanically stable LESC cultivation while maintaining a low scaffold thickness. PDLLA electrospun membranes have a degradation timeframe of at least 5 months or more [44,45]. As described elsewhere, almost two months were needed for long-term cultivation of differentiated retinal cell monolayer on polylactide membrane for subretinal implantation. Even after two months, the nanofibrous scaffold kept its shape and allowed for stable protection of the cultivated cells, loading through the implantation injector, and delivering to the subretinal space during surgery [46]. Our ultrathin nanofibrous membrane possessing a low thickness of 4.1 μm could allow both oxygen and nutrient absorption to the underlying ocular surface; a graft would be completely covered by tear film, which is 4–7 μm thick [30]. Unlike a difficult surgical manipulation with the fragile fibrin matrix, the incorporation of a supporting frame can allow for addressing possibly problematic manipulation with wet electrospun membranes, as described in our previous study [47]. Ultrathin nanofibrous membranes equipped with preferably degradable supporting structures allow for delivering the cell graft without folding the sample or damaging the cell layer. The optical transparency of the wet PDLLA membrane (similar to that of the fibrin gel) could allow eyelid function and ocular surface inflammatory status control in clinical use. Notably, the relatively high transmittance was satisfactory compared to that of HAM, which was reported to possess transmittances of 38 % and 27 % at 700 nm and 400 nm, respectively [23].

While the physicochemical characterization of the fabricated membranes proved the presence of fibronectin, negligible changes in the fibre morphology were found by SEM (Fig. 1C). These were most probably caused by the fusion of the polymer, as induced by the sterilization procedure, being in line with the previously published works [[48], [49], [50]]. Regarding the XPS characterization, we noted the consistent quantitative observations in the high-resolution spectra taken in the C 1s and N 1s regions, pointing to low variations of the determined amide contributions originating from the fibronectin deposited on the PDLLA membrane (Table 2). This, therefore, points to the homogeneous distribution of the protein over the PDLLA fibrous scaffold.

Regarding LEC cultivation, our data indicate that PDLLA membranes, similar to fibrin gel, support the cultivation of primary LECs. The resulting PDLLA-based cultures, however, differed morphologically from those grown on fibrin gel. Multicellular structures and cell networks showing heterogeneous morphological appearance with the presence of spindle-shaped cells were found after extended cultivation time (16–21 days). The finding that extended cultivation time could lead to morphological differences was supported by the work of Polisetti and coworkers [51]. In their study, spindle cells, referred to as mesenchymal cells of the limbus, were present in extended limbal explant cultures on the de-epithelialized HAM.

Mariappan and coworkers reported similar observations [52]; secondary to close stem cell and niche cell interactions with each other, *ex vivo* cultivation of limbal suspension cultures led to cell self-organization to form three-dimensional structures and whorled growth patterns (cf. Fig. 4C and D), emphasizing limbal crypt-related processes. The base of limbal crypts serves as a protected microenvironment for LESCs close to a variety of neighbouring niche cells [53]. Biomaterial scaffold micropatterning and preparation of artificial stem cell niches resembling the limbal crypts have been shown to provide a unique microenvironment for mechanical support and biological signals and to modulate in vivo cell behaviour [24,53,54]. Considering these previous findings along with our observations indicates that the micropattern of the studied nanofibrous PDLLA membrane serves as a microenvironment allowing biological communication and modulating cell expansion and the resulting phenotype. In other words, the inclusion of the electrospun PDLLA nanofiber-based pattern provides the cells with a physically protected space, a feature that is not possessed by fibrin gel. The preferential self-organization of PDLLA membrane cultures emphasizes a need for further in vivo testing to shed light on the regenerative potential of PDLLA culture.

Furthermore, the gene expression analysis revealed that cells obtained under the two culture conditions had several differences. Regarding the stemness, cells cultured on fibrin gels exhibited significantly higher expression of the stem cell markers *OCT4* and *NGFR,* while PDLLA-cultured samples had higher expression of *LRIG1*. POU domain class 5, transcription factor 1 (OCT4) and tumour necrosis factor receptor superfamily member 16 (NGFR) are associated with ocular surface epithelial stem/progenitor cells [55]. In contrast, leucine-rich repeats and immunoglobulin-like domains 1 (LRIG1) has emerged as a key regulator of stem cell behaviour due to its function in growth factor receptor regulation [56], and its higher expression is associated to stem cell quiescence through bone morphogenetic protein signalling [57]. These findings suggest that fibrin gel cultures retained a higher expression of stem cell and progenitor cell potential.

We also found higher mesenchymal cell/fibroblast marker gene expression in the case of PDLLA membrane cultures. Alpha-actin-2 (ACTA2), a myofibroblast-specific marker, has been linked to the epithelial-to-mesenchymal transition (EMT) [58]. The higher expression of *ACTA2* in cells cultured on the PDLLA scaffolds might be aligned with the observed heterogeneity in cell morphology and the formation of self-organizing structures on the PDLLA membrane, whereas on fibrin, the cells showed a cobblestone epithelial morphology. Similarly, *THY1* expression, which is exclusive to corneal fibroblasts and myofibroblasts (but not keratocytes) [59], might indicate the presence of mesenchymal cells/fibroblasts. It is worth stressing that in the present study, the PDLLA membrane surface was coated with fibronectin, which is known to facilitate epithelial adhesion and migration [60,61]. On the other hand, fibronectin was shown to promote a pro-mesenchymal cell phenotype, as demonstrated by Camara and coworkers, who showed that fibronectin influences EMT in primary human bronchial epithelial cells [62]. As observed in the gene expression of PDLLA membrane-based cultures, the presence of fibronectin could presumably modulate the mesenchymal cell/fibroblast phenotype. The specific mechanisms and signalling pathways involved in this process of differentiation of limbal epithelial cells towards corneal fibroblasts require further investigation.

On the other hand, differentiation to epithelial cells was present in both PDLLA membrane- and fibrin gel-based cultures, although *KRT14* was significantly upregulated in fibrin gel cultures. *KRT14* and *KRT15* are used to identify LESCs/early progenitor cells [20,51,63,64]. We found high expression of *KRT7*, demonstrated by both RT–qPCR and immunofluorescence. LESCs were reported for *KRT7* upregulation by Albert and coworkers [65], but this marker has also been widely used for differentiated corneal epithelial and conjunctival epithelial cells. Additionally, both culture conditions had comparable levels of *KRT12* expression, indicating that limbal stem cells underwent differentiation towards the corneal phenotype, as keratin 12 is a cell type-specific protein characteristic of differentiated corneal epithelium [66].

Notably, there was interindividual variation in the expression of some stem cell markers, indicating the influence of the donor on the results of the culture regardless of the biomaterial employed. In our study, a group of donors had relatively higher levels in the stem cell markers *KLF4*, *ΔNp63α* and *ABCG2* and keratins 14, 15, and 7. Krüppel-like Factor 4 (KLF4) plays a crucial role in inhibiting epithelial-to-mesenchymal transition in human corneal epithelial cells, preventing the development of fibrotic scars in the cornea by reducing the activity of TGF-β signalling [67]. ΔNp63α is a distinct marker for stem cells located within the basal layer of the limbus [68]. ATP-binding cassette, subfamily G, member 2 (ABCG2) has been proposed as a universal and conserved marker for stem cells from various tissues [69]. In another study [70], cells of the smallest size (10–16 μm) expressed the highest levels of the putative stem cell markers ΔNp63 and ABCG2 at both mRNA and protein levels. They also contained the highest number of label-retaining cells (side population) and had the highest clonogenic capacity in a culture. Our donor sample was relatively homogeneous, although we cannot exclude differences in phenotypes and genetic background. These results imply that the effect of the donor in the stemness and LECs differentiation is important and merits further study.

In summary, cultures on fibrin gel retained a higher percentage of cells expressing stem cell or progenitor cell markers than cultures on PDLLA membrane. Both culture conditions, however, exhibited the presence of these markers to some extent. Furthermore, cells cultured on the PDLLA membrane showed upregulation of genes associated with the mesenchymal phenotype, suggesting a potential shift towards a stromal phenotype, while cells on fibrin gels displayed increased expression of genes related to corneal epithelial differentiation. Further studies are warranted to envision the regenerative potential of LECs originating from PDLLA cultures.

We believe that this fundamental study will pave the way for the consideration of polymeric nanofibrous scaffolds in the study design of eye regenerative applications. To find an appropriate culture condition to produce cell cultures possessing high stemness capacity, studies focused on LEC phenotype changes over different harvesting/culturing times are indicated.

## 5 Conclusion

We prepared, characterized, and comprehensively studied PDLLA nanofibrous membranes. In comparison to the mechanically fragile fibrin gel, ultrathin PDLLA-based membranes provide a stable substrate for LEC cultivation while maintaining high optical transparency. The fabricated scaffolds effectively supported the *ex vivo* cultivation of LECs. When compared to cultivation on fibrin gel, LECs grown on PDLLA nanofibrous scaffolds exhibited variations in cell expansion and morphology, while still maintaining similar expression of LEC and stemness markers. The morphology of cells cultured on PDLLA did not clearly resemble epithelial cells, unlike those cultured on fibrin gel. The presence of mesenchymal cells/fibroblasts was consistent with the gene expression, as significantly higher expression of mesenchymal/fibroblast markers was observed in PDLLA cultures. Overall, the PDLLA nanofibrous scaffolds demonstrate potential for application in the field of regenerative medicine of the eye and contribute to the development of a limbal cell culture protocol in which both carrier materials are xeno-free, highly defined, and fully standardized.

## Ethical approval

The study adhered to the tenets set out in the Declaration of Helsinki; donor tissue procurement met all Czech legal requirements, including the absence of the donor in the national register of persons opposed to post-mortem withdrawal of tissues and organs. Based on the Czech legislation on specific health services (Law Act No. 372/2011 Coll.), informed consent is not required if the presented data are anonymized in the form. Limbal cell isolation from deceased human donors was approved by the General University Hospital in Prague, Czech Republic.

## Data availability statement

Data included in article/supp. material/referenced in article.

## CRediT authorship contribution statement

**Jiří Trousil:** Writing – original draft, Validation, Methodology, Investigation, Data curation, Conceptualization. **Joao Victor Cabral:** Writing – original draft, Validation, Methodology, Investigation, Data curation, Conceptualization. **Eleni Voukali:** Writing – original draft, Formal analysis, Data curation. **Jitka Nováčková:** Formal analysis. **Ognen Pop-Georgievski:** Formal analysis, Writing – original draft. **Tomáš Vacík:** Data curation. **Pavel Studený:** Investigation. **Hana Studenovska:** Writing – original draft, Validation, Supervision, Methodology, Investigation, Conceptualization. **Katerina Jirsova:** Writing – original draft, Validation, Supervision, Methodology, Investigation, Conceptualization.

## Declaration of competing interest

The authors declare that they have no known competing financial interests or personal relationships that could have appeared to influence the work reported in this paper.
